# Use of red grape pomace and Aloe vera gel as nutraceuticals to ameliorate stocking density-induced stress in commercial male broilers

**DOI:** 10.1007/s11250-024-03943-x

**Published:** 2024-03-20

**Authors:** Kwena Kgaogelo Thema, Victor Mlambo, Chidozie Freedom Egbu, Caven Mguvane Mnisi

**Affiliations:** 1 Food Security and Safety Focus Area, Faculty of Natural and Agricultural Science, North-West University, Private Bag x2046, Mmabatho, 2735 South Africa; 2 School of Agricultural Sciences, Faculty of Agriculture and Natural Sciences, University of Mpumalanga, Mbombela, 1200 South Africa

**Keywords:** Feed additive, Growth performance, Oxidative stress, Phytochemicals, Poultry welfare

## Abstract

The combined effect of Aloe vera gel (AVG) administered through drinking water and dietary red grape pomace powder (RGP) on growth performance, physiological traits, welfare indicators, and meat quality in densely stocked broilers was evaluated. A total of 750, two-week-old male Ross 308 broilers (317.7 ± 10.12 g live weight) were randomly assigned to 25 cages, with each cage as an experimental unit. The broilers were stocked at a density of 30 birds per cage with a floor space of 1.32 m^2^. Dietary treatments were a standard grower or finisher diet (CON); CON containing 30 g RGP /kg diet plus either 1 (GPA1), 2 (GPA2), 3 (GPA3), or 4% (GPA4) AVG in drinking water. Treatment GPA1 promoted higher (*P* < 0.05) overall weight gain and overall feed conversion ratio (FCR) than CON. Positive quadratic effects (*P* < 0.05) were noted for mean corpuscular hemoglobin, basophils, 24-hour breast meat yellowness, chroma, and hue angle. The GPA2 group had the lowest (*P* < 0.05) gait score while the CON group had the highest score. Concurrent supplementation with a 30 g RGP /kg diet plus 1% AVG in drinking water enhanced weight gain, FCR, and finisher weight of densely stocked broilers. However, AVG doses beyond 1% did not enhance performance and physiological traits in densely stocked broilers.

## Introduction

High-input poultry production systems involve the confinement of chickens at high stocking densities to maximize profit with little regard for bird welfare (Tainika et al., 2023). This practice has been met with public displeasure from poultry consumers worldwide (Jobe et al., 2019; Escobedo del Bosque et al., 2021). In addition, high stocking densities result in poor feed utilization efficiency, cannibalism, and weakened immune systems, which ultimately affect growth performance (Thema et al., 2022). High stocking density (SD) in broilers induces a combination of physical, environmental, and social stress factors that disrupt the physiological balance and antioxidant defense systems of the chickens (Bilal et al., 2021; Hafez et al., 2022). This causes an upsurge in the synthesis of reactive oxygen species and a decline in their neutralization, ultimately leading to oxidative stress (Jobe et al., 2019; Sumi et al., 2019). Nutraceutical plants contain potent antioxidant properties that can alleviate oxidative stress and improve growth performance, gut health, and poultry welfare (Alloui et al., 2014; Yadav et al., 2020). Consequently, natural sources of bioactive substances with growth- and health-promoting effects such as red grape pomace (RGP; *Vitis vinifera* L.) and Aloe vera gel (AVG; *Aloe barbadensis* M.) have attracted worldwide research attention (Ebrahimzadeh et al., 2018; Jalal et al., 2019; Sumi et al., 2019).

Red grape pomace is a waste by-product generated during grape juice or wine production, comprising the seeds, skin, and stems (Makri et al., 2017). It is rich in phenolic compounds such as procyanidins, epicatechin-3-O-gallate, epicatechins, and catechins (Rockenbach et al., 2011; Van Niekerk et al., 2020) that have been shown to exhibit antibacterial (Oliveira et al., 2013) and antioxidant activities (Makri et al., 2017). Moreover, RGP has been shown to stimulate growth (Viveros et al., 2011), enhance the immune system (Ebrahimzadeh et al. 2018), and have anti-lipidemic effects (Hosseini-Vashan et al., 2020) in broiler chickens.

On the other hand, Aloe vera is a shrubby succulent plant that contains biomolecules such as acemannan, aloesin, emodin, and aloin that have immunomodulatory and antibacterial effects (Sánchez et al., 2020). These biomolecules have been shown to boost bird immunity (Darabighane and Nahashon, 2014; Ahmad et al., 2020). Furthermore, AVG contains polyphenols and natural antioxidants that neutralize free radicals implicated in lipid peroxidation in animal models (Sharma et al., 2018). Although positive results have been reported when RGP (Kumanda et al., 2019) and AVG (Jalal et al., 2019) were separately used as dietary additives, their possible synergistic effects in broiler chickens have not been investigated. The RGP and AVG may have complementary effects on stressed broilers due to differences in the profiles of their bioactive compounds (Rockenbach et al., 2011; Kumar and Tiku, 2016). Additionally, RGP and AVG have prebiotic effects (Yu and Ahmedna, 2013; Zhu et al., 2015) that may enhance gut health leading to improved nutrient absorption, growth, and physiological performance. This study, therefore, tested the effect of combining a fixed level of RGP (30 g/kg) with 1, 2, 3, and 4% AVG on growth performance, physiological responses, welfare indicators, and meat quality parameters of densely stocked broiler chickens (30 birds/cage).

## Materials and methods

### Ingredients and diet formulation

The RGP was bought from Blaauwklippen Wine Estate (Stellenbosch, South Africa), air-dried to constant weight, and thereafter milled (1 mm). The RGP contained 91.78% dry matter (DM), 11.67% DM crude protein, 36.19% DM neutral detergent fibre, 30.2% DM acid detergent fibre, 17.79% DM acid detergent lignin and 3.22% DM ether extract as described in our previous work (Mhlongo et al., 2022). The AVG was purchased from Forever Living Products (Westfield, South Africa). According to the manufacturer, the AVG consisted of polysaccharides (55%), proteins (7%), lipids (4%), minerals (16%), sugars (17%), ascorbic acid (0.2%), phenolic compounds (1%), sodium benzoate (0.1%), tocopherol (0.004%), sorbitol (3.2%), potassium sorbate (0.1%), and xanthan gum (0.02%).

The experimental diets (Table 1), in a mash form, comprised the grower (14–28 days of age) and finisher (29–42 days of age) phases to satisfy or surpass the nutritional requirements of Ross broiler chickens (Aviagen, 2014). The five treatment groups were: (1) a standard chicken diet (CON) without AVG in drinking water, (2) CON containing 30 g RPG /kg diet plus 1% AVG in drinking water (GPA1), (3) CON containing 30 g RPG /kg diet plus 2% AVG in drinking water (GPA2), (4) CON containing 30 g RPG /kg diet plus 3% AVG in drinking water (GPA3), and (5) CON containing 30 g RPG /kg diet plus 4% AVG in drinking water (GPA4). Throughout the feeding trial, the broilers were given unlimited access to fresh water and feed. The calculated composition of the test diets is shown in Table 1.

**Table 1:** Ingredient composition (g/kg as fed) of experimental diets

Ingredients	Grower (14–28 days)		Finisher (29–42 days)	
	CON	GP30	CON	GP30
Red grape pomace	0.00	30.0	0.00	30.0
Soybean oilcake (46.5%)	199	124	168	98.0
Full-fat soybean	42.0	138	55.0	140
Corn gluten 60	21.0	24.0	0.00	0.00
Lysine (sint 78%)	2.90	2.90	1.90	1.83
Methionine (dL 98%)	1.90	1.70	1.50	1.35
Threonine (98%)	0.30	0.30	0.10	0.06
Maize yellow	704	650.5	750.8	706.36
Feed lime (50:50 mix)	14.5	14.0	12.5	12.2
MDCP (ws > 70%)	7.20	7.40	2.20	2.30
Salt (fine)	3.10	3.20	2.80	2.90
Sodium bicarbonate	1.80	1.70	1.90	1.70
Axtraphytase (100 g/t sk)	0.10	0.10	0.10	0.10
Zinc bacitracin (15%)	0.00	0.00	0.50	0.50
Choline Cl (60%)	0.80	0.80	0.00	0.00
Salinomycin (12%)	0.50	0.50	0.00	0.00
Olaquindox (10%)	0.40	0.40	0.20	0.20
^#^Premix no spec	0.50	0.50	0.00	0.00
^#^Premix no spec + choline chloride	0.00	0.00	2.50	2.50
Total	1000	1000	1000	1000
Calculated composition (g/kg as is, unless mentioned otherwise)				
Dry matter	892	896	890	893
^*^Metabolizable energy (MJ/kg)	12.0	12.0	12.1	12.1
Crude protein	170	170	160	161
Crude fat	34.2	35.4	42.0	53.2
Crude fiber	25.0	38.6	34.2	47.1
Organic matter	822	847	846	852
Calcium	7.99	8.10	6.65	6.60
Phosphorus	4.99	4.91	3.30	3.33
Sodium	1.80	1.80	1.80	1.80
Chloride	2.88	3.10	2.50	2.50
Potassium	7.21	7.10	6.65	6.60

^1^Diets: CON = a standard chicken diet without red grape pomace and Aloe vera gel in drinking water; GP30 = a standard chicken diet containing 3% red grape pomace

^#^Premix no spec was purchased from Nutroteq (Centurion, South Africa)

^*^Metabolizable energy values were predicted using models from near infrared reflectance spectroscopy (SpectraStar XL, Unity Scientific, Australia)

### Experimental design, feeding, and broiler management

The feeding experiment was carried out at Molelwane research farm (26º41’36” S, 27º05’35” E) in Mafikeng, South Africa. In a single house, seven hundred and fifty (750) day-old Ross 308 male chicks were raised using a conventional starter mash diet from NutriFeeds (Lichtenburg, South Africa). This feeding lasted for 10 days, from which the birds were accustomed to the experimental diets for three days. On day 14, the birds were randomly distributed into 25 cages based on body weight, with each cage serving as an experimental unit. Our preliminary study revealed that Ross 308 broilers can be stocked at a density of 20 birds per cage of 1.32 m^2^ floor space (Thema et al., 2022). Thus, in this study, a higher stocking density was used with each replicate cage carrying 30 birds. The treatments were replicated 5 times following a completely randomized design. The cages measured 1.1 m length × 1.2 m width × 1.55 m height, providing a floor space of 1.32 m² including the area occupied by 5L tube feeders and 5L drinkers. The floor of the cages was covered with polythene sheets, which were cleaned regularly. During the initial two weeks, infrared electric lights were utilized to keep the temperature at 34 °C, which was then dropped by 2°C every other week until 28 °C. The house was ventilated by opening curtains in the morning (06h00) and closing them in the evening (18h00). The feeding trial was conducted under natural lighting.

### Growth performance

Feed intake (FI) by the birds was measured daily as the difference between the provided feed and feed remaining. After measuring initial body weights (317.7 ± 10.12 g live body weight) on day 14, the live weights of the birds were measured weekly until day 42. The live weight data was used to calculate body weight gain (BWG) per bird. The data for the average FI and the average BWG were computed to determine feed conversion ratio (FCR). Mortality was recorded on occurrence and the dead birds were replaced with broiler chickens of similar weights (raised in groups fed the same diets) so as not to affect the SD per cage. The FCR data was calculated and adjusted for mortalities.

### Blood collection and examination

On day 40, 10 birds per treatment were randomly picked from every replicate cage for blood sampling. The brachial vein was used to sample at least 4 mL of fresh blood using disposable syringes and needles, which was then promptly transferred into whole blood (with EDTA) and serum tubes. Subsequently, an automated LaserCyte Hematology Analyzer (IDEXX Laboratories SA Pty, Midrand, South Africa) was utilized to determine the concentration of erythrocytes, hematocrits, hemoglobin, neutrophils, mean corpuscular volume (MCV), red cell distribution width (RDW), mean corpuscular hemoglobin (MCH), basophils, reticulocytes, lymphocytes, white blood cell (WBC), eosinophils, monocytes, and platelets. Blood samples from the serum tubes were centrifuged at 3500 rpm for 15 min and the serum was used to analyze for albumin, glucose, alanine transaminase (ALT), calcium, symmetrical dimethylarginine (SDMA), total cholesterol, phosphorus, globulin, total protein, lipase, amylase, and alkaline phosphatase (ALKP) using an automated Vet Test Chemistry Analyzer (IDEXX Laboratories SA Pty, Midrand, South Africa).

### Welfare indicators

On day 41, a random selection of two birds per cage was done for the latency-to-lie test (LTL) following the protocol by Berg and Sanotra (2003). Each bird was placed in a plastic water container with water filled to a height of 3 cm at 32 °C. The duration taken by the bird to lie was recorded. Broilers that had shorter standing durations were perceived to have weak legs, while those that had longer standing durations were perceived to have strong legs. In a case where the bird stood for more than 10 min, the test was stopped, and the legs were deemed healthy and strong. For gait score tests, the birds’ natural walking movements were observed, and their walking abilities were rated on a scale of 0 to 5, following the protocol established by Van Hertem et al. (2018). A score of 0 indicated a normal gait or free walking, while a score of 5 indicated that the bird was unable to walk. Feather score was determined using the protocol by Bilcik and Keeling (1999), where each bird was gently stroked along the keel bone from the front to the back using the palm of the hand. The level of exposed flesh visible through the compressed feathers were subjectively rated on a scale of 3 points: 1 – denoted complete coverage of feathers with no visible skin, 2 – indicated a relatively small amount of exposed skin, and 3 – indicated a relatively large amount of exposed skin.

### Carcass characteristics and internal organs

On day 42, every chicken in a cage was weighed to determine average final body weight (FBW) per cage. The broilers were then packed in crates and driven to a local abattoir for slaughter after being stunned. Bleeding was allowed for at least 2 min and thereafter, the carcasses were all de-feathered and manually eviscerated. The individual weights of the carcasses were immediately recorded after slaughter as hot carcass weight (HCW) and then chilled at 4 °C for 24 h to record cold carcass weight (CCW). Carcass yield was calculated by expressing the HCW as a proportion of FBW. Individual cold carcasses per cage were used to determine the weights of carcass retail cuts (thigh, wing, drumstick, and breast), and internal organs (proventriculus, spleen, liver, gizzard, duodenum, jejunum, ileum, caecum, and colon, along with their contents).

### Breast meat quality measurements

Breast meat pH was measured from all the carcasses using a pH meter (HI98163, Hanna Instruments, Woonsocket, RI, USA), which was calibrated using standard pH solutions (4, 7, and 10) provided by the supplier. The surface of the breast muscle from all the carcasses was assessed for redness (a*), yellowness (b*), and lightness (L*) using a colorimeter (Minolta BYK-Gardener GmbH, Geretsried, Germany) post-slaughter. The colorimeter was set to read at a 20-mm diameter measurement area (aperture size), illuminant D65-day light, and 10° observation angle. Cooking loss was determined by weighing the raw breast muscle and then cooking to reach an inner temperature of 75 °C (Honikel, 1998). Raw breast meat samples were sheared using a Meullenet-Owens Razor Shear Blade (A/MORS) attached to a Texture Analyzer (TA.XT plus, Stable Micro Systems, Surrey, UK) to measure peak shear force (N) values. The water-holding capacity (WHC) of the breast meat was determined using the filter-paper press method developed by Grau and Hamm (1953). Drip loss was evaluated by suspending pieces of breast muscle weighing 80–120 g in a chilled environment (1–5 °C) for 72 h before re-weighing to calculate drip loss following the procedure outlined by Honikel (1998).

### Statistical analysis

Data for weekly FI, BWG, and FCR were analyzed using repeated measures analysis in PROC GLM (SAS, 2013) to assess the interaction effect of diet and time (chicken age in weeks) using the following model:

$${Y}_{ijk} = \mu +{D}_{i}+{W}_{j}+{\left(D\times W\right)}_{ij}+ {E}_{ijk}$$

where, *Y*_*ijk*_ = response variables, *µ* = population mean, *D*_*i*_ = dietary treatment effects, *W*_*i*_ = time (in weeks), *(*$D\times$*W)*_*ij*_ = the effect of interaction between dietary treatment and weeks, and *E*_*ijk*_ = random error associated with observation *ijk*, assumed to be normally and independently distributed. Performance, physiological responses, welfare and meat quality data were also analyzed using a one-way analysis of variance (ANOVA) by means of PROC GLM in SAS, where treatment was the main factor. The least-squares means were then distinguished using the probability of difference options in SAS. Moreover, the data (except for CON data) were tested for using polynomial contrasts. Further, response surface regression analysis (PROC RSREG) was applied to determine linear and quadratic effects in response to different levels of AVG (1–4%), assuming that the RGP effect was fixed. Since the welfare parameters did not meet the normality assumption of a one-way ANOVA, the Kruskal–Wallis test was used to determine the treatment effect on gait and feather scores in the chickens (MacFarland et al., 2016). The level of significance was set at *P* < 0.05 for every measurement.

## Results

### Growth performance

There were no significant week × treatment interaction effects on FI (*P* = 0.221), BWG (*P* = 0.054), and FCR (*P* = 0.053). Table 2 shows that no linear or quadratic responses (*P* > 0.05) were recorded for overall FI as AVG levels were increased. However, increasing AVG doses in drinking water linearly reduced overall BWG [*y* = 1584 (± 65.7) – 24.6 (± 59.5) *x*; R^2^ = 0.528, *P* = 0.002] and FCR [R^2^ = 0.332, *P* = 0.026]. Similarly, birds reared on GPA1 had higher (*P* < 0.05) overall BWG than those reared on CON and GPA4 treatments. The GPA1 treatment resulted in higher (*P* < 0.05) overall FCR than the CON, GPA2, and GPA4 treatments.

**Table 2:** Overall growth performance (day 14–42) in densely stocked broiler chickens offered a red grape pomace-containing diet and incremental levels of Aloe vera gel in their drinking water

^2^Parameters	^1^Treatments						Significance		
	CON	GPA1	GPA2	GPA3	GPA4	^3^SEM	*P*-_GLM_	*P*-_Linear_	*P*-_Quadratic_
FI (g/bird)	3025	3078	3114	3064	2983	39.5	0.204	0.068	0.215
BWG (g/bird)	1404^a^	1556^c^	1505^bc^	1516^bc^	1431^ab^	22.4	0.001	0.002	0.798
FCR (g:g)	2.155^a^	1.978^c^	2.069^ab^	2.021^bc^	2.084^ab^	0.010	0.001	0.026	0.394

^a,b,c^Means with distinct superscripts within the same row indicate significant differences (*P* < 0.05)

^1^Treatments: CON = standard chicken diet without red grape pomace and no Aloe vera gel in drinking water; GPA1 = standard chicken diet containing 30 g/kg red grape pomace and 1% Aloe vera gel in drinking water; GPA2 = standard chicken diet containing 30 g/kg red grape pomace and 2% Aloe vera gel in drinking water; GPA3 = standard chicken diet containing 30 g/kg red grape pomace and 3% Aloe vera gel in drinking water; GPA4 = standard chicken diet containing 30 g/kg red grape pomace and 4% Aloe vera gel in drinking water

^2^Parameters: FI = feed intake; BWG = body weight gain; FCR = feed conversion ratio

^3^SEM: standard error of the mean

### Blood parameters

There were positive quadratic effects for MCH [*y* = 5.03 (± 1.24) *x*^2^ – 27.8 (± 6.34) *x* + 64.9 (± 7.25); R^2^ = 0.635, *P* = 0.002] and basophil [*y* = 0.475 (± 0.152) *x*^2^ – 2.79 (± 0.779) *x* + 4.52 (± 0.890); R^2^ = 0.595, *P* = 0.009] as AVG doses in drinking water increased (Table 3). However, erythrocytes showed a negative quadratic effect [*y* = 3.57 (± 0.863) *x* – 0.629 (± 0.169) *x*^2^ − 1.67 (± 0.986); R^2^ = 0.628, *P* = 0.003] on the AVG doses. There were significant treatment effects on erythrocytes, SDMA, phosphorus, and amylase. The birds on treatment GPA1 had the least (*P* < 0.05) erythrocyte counts and SDMA than all the other groups. The GPA4 promoted the highest phosphorus than GPA1 and GPA3 but had statistically similar serum phosphorus levels as CON and GPA2 treatments. Birds on GPA2 had higher (*P* < 0.05) amylase levels than GPA1 and GPA3 but did not vary (*P* > 0.05) with the other groups.

**Table 3:** Blood parameters of densely stocked Ross 308 broilers (*n* = 50) offered a red grape pomace-containing diet and incremental levels of Aloe vera gel in their drinking water

^2^Parameters	^1^Treatments						Significance		
	CON	GPA1	GPA2	GPA3	GPA4	^3^SEM	*P-*_GLM_	*P*-_Linear_	*P*-_Quadratic_
Erythrocytes (×l0^12^/L)	2.66^b^	1.39^a^	3.19^b^	3.28^b^	2.73^b^	0.35	0.007	0.027	0.003
Hematocrits (L/L)	16.0	18.5	19.7	22.4	17.7	2.02	0.261	0.959	0.403
Hemoglobin (g/dL)	8.96	8.29	9.00	8.62	8.40	0.25	0.247	0.521	0.377
MCV (fL)	58.6	59.1	61.3	59.1	64.9	4.36	0.829	0.539	0.302
MCH (pg)	34.6	33.5	33.2	32.2	34.1	3.83	0.046	0.058	0.002
RDW (×10^9^/L)	25.5	24.8	27.1	24.7	24.3	1.52	0.722	0.806	0.133
Reticulocytes (K/µL)	325	96.4	218	320	418	125	0.448	0.141	0.456
WBC (×10^9^/L)	120	99.0	34.5	84.2	73.4	36.5	0.561	0.620	0.967
Neutrophils (×10^9^/L)	11.6	28.0	1.19	10.9	3.18	9.58	0.333	0.991	0.923
Lymphocytes (×10^9^/L)	90.9	53.2	29.0	62.3	57.8	27.6	0.636	0.415	0.730
Monocytes (×10^9^/L)	6.46	9.93	1.20	5.00	2.77	3.11	0.349	0.977	0.698
Eosinophils (×10^9^/L)	12.5	10.6	8.36	10.4	11.0	1.60	0.508	0.465	0.356
Basophils (×10^9^/L)	1.36	1.53	0.44	0.82	0.76	0.31	0.126	0.016	0.009
Platelets (×10^9^/L)	2165	1033	1567	1880	1248	467	0.448	0.266	0.646
Glucose (mmol/L)	8.65	8.81	9.50	9.31	9.35	0.26	0.119	0.395	0.637
SDMA (µg/dL)	50.0^b^	40.5^a^	49.5^b^	51.2^b^	50.2^b^	2.25	0.018	0.194	0.279
Phosphorus (mmol/L)	2.38^ab^	2.36^a^	2.45^ab^	2.32^a^	2.54^b^	2.38	0.014	0.390	0.147
Calcium (mmol/L)	2.43	2.39	2.33	2.43	2.45	2.43	0.942	0.699	0.358
Total protein (g/L)	38.4	41.5	38.9	40.0	40.2	0.94	0.198	0.884	0.590
Albumin (g/L)	0.54	0.53	0.49	0.50	1.06	0.25	0.457	0.479	0.117
Globulin (g/L)	26.0	27.9	27.2	27.4	27.1	0.94	0.700	0.583	0.659
ALT (U/L)	31.6	30.6	27.6	32.1	29.3	2.28	0.457	0.715	0.910
ALKP (U/L)	328	262	275	323	279	31.0	0.462	0.523	0.443
Total Cholesterol (mmol/L)	3.12	3.09	3.26	3.07	3.05	0.10	0.564	0.703	0.128
Amylase (U/L)	342^bc^	261^a^	377^c^	295^ab^	343^bc^	24.8	0.029	0.325	0.312
Lipase (U/L)	163	139	156	145	132	14.2	0.557	0.734	0.273

^a,b,c^Means with distinct superscripts within the same row indicate significant differences (*P* < 0.05)

^1^Treatments: CON = standard chicken diet without red grape pomace and no Aloe vera gel in drinking water; GPA1 = standard chicken diet containing 30 g/kg red grape pomace and 1% Aloe vera gel in drinking water; GPA2 = standard chicken diet containing 30 g/kg red grape pomace and 2% Aloe vera gel in drinking water; GPA3 = standard chicken diet containing 30 g/kg red grape pomace and 3% Aloe vera gel in drinking water; GPA4 = standard chicken diet containing 30 g/kg red grape pomace and 4% Aloe vera gel in drinking water

^2^Parameters: MCV = mean corpuscular volume; MCH = mean corpuscular hemoglobin; RDW = red cell distribution width; WBC = white blood cells; ALT = alanine transaminase; ALKP = alkaline phosphatase; SDMA = symmetric dimethylarginine

^3^SEM: standard error of the mean

### Welfare indicators

Table 4 shows that the LTL linearly increased [*y* = 22.2 (± 72.4) *x* + 160 (± 61.1); R^2^ = 0.306, *P* = 0.0001] with the increasing AVG doses. Similarly, the LTL test was affected (*P* < 0.05) by treatments where the CON group had the shortest (159.03 s) and the GPA4 group had the longest (529.87 s) times. A Kruskal-Wallis H test showed that there was a significant (*P* < 0.05) impact on gait score [χ^2^(2) = 9.962, *P* < 0.041]. The medians were 31.2, 33.3, 18.7, 23.3, and 21.0 for CON, GPA1, GPA2, GPA3, and GPA4, respectively. Kruskal-Wallis H test also showed that there was no significant treatment effect on feather scores.

**Table 4:** Welfare indicators in densely stocked broiler chickens offered a red grape pomace-containing diet and incremental levels of Aloe vera gel in their drinking water

Parameters	^1^Treatments							Significance	
	CON	GPA1	GPA2	GPA3	GPA4	SEM	*P-*_value_	*P*-_Linear_	*P*-_Quadratic_
Latency-to-lie (sec)	159^a^	196^a^	287^ab^	366^ab^	530^b^	66.29	0.002	< 0.0001	0.326
Gait score*	31.2	33.3	18.7	23.3	21.0	-	0.041	-	-
Feather score*	32.5	25.0	25.0	22.5	22.5	-	0.344	-	-

^1^Treatments: CON = standard chicken diet without red grape pomace and no Aloe vera gel in drinking water; GPA1 = standard chicken diet containing 30 g/kg red grape pomace and 1% Aloe vera gel in drinking water; GPA2 = standard chicken diet containing 30 g/kg red grape pomace and 2% Aloe vera gel in drinking water; GPA3 = standard chicken diet containing 30 g/kg red grape pomace and 3% Aloe vera gel in drinking water; GPA4 = standard chicken diet containing 30 g/kg red grape pomace and 4% Aloe vera gel in drinking water

* Gait and feather scores represent the medians and the chi-square *P*-values from the Kruskal-Wallis test

### Carcass characteristics and visceral organs

There were no linear or quadratic trends (*P* > 0.05) for carcass traits and visceral organs, except for FBW which increased linearly [*y* = 15.0 (± 57.3) *x* + 1852 (± 62.8); R^2^ = 0.284, *P* = 0.022] with AVG doses (Table 5). Treatment GPA1 promoted the highest FBW followed by GPA3, GPA2, and GPA4 while the lowest FBW was from birds reared on the CON. However, the FBW of birds reared on CON and GPA4 did not vary (*P* > 0.05).

**Table 5:** Carcass characteristics and internal organs in densely stocked Ross 308 broiler chickens offered a red grape pomace-containing diet and incremental levels of Aloe vera gel in their drinking water

^2^Parameters	^1^Treatments						Significance		
	CON	GPA1	GPA2	GPA3	GPA4	^3^SEM	*P*-_GLM_	*P*-_Linear_	*P*-_Quadratic_
Dressing (%)	75.0	73.3	73.8	71.9	73.8	1.81	0.825	0.990	0.676
FBW (g)	1757^a^	1867^d^	1824^bc^	1850^cd^	1775^ab^	23.0	0.012	0.022	0.485
HCW (g)	1317	1369	1346	1331	1311	35.9	0.790	0.243	0.963
CCW (g)	1305	1359	1333	1317	1302	35.6	0.784	0.247	0.879
Wing (g)	78.6	77.7	78.8	80.3	83.4	3.22	0.753	0.113	0.789
Drumstick (g)	86.4	85.0	90.9	89.8	84.0	3.87	0.666	0.726	0.423
Thigh (g)	99.8	102	99.9	99.6	108	0.18	0.046	0.081	0.260
Breast (g)	259	248	249	253	242	10.6	0.832	0.100	0.687
Proventriculus (g)	6.97	7.96	7.41	7.26	7.52	0.48	0.686	0.354	0.097
Spleen (g)	2.08	2.06	2.08	2.10	1.99	0.13	0.972	0.635	0.656
Liver (g)	40.9	43.5	43.8	42.3	45.4	1.33	0.193	0.399	0.487
Gizzard (g)	33.0	34.8	36.4	35.1	36.2	0.88	0.084	0.219	0.916
Duodenum (g)	14.1	14.9	13.4	14.0	14.7	0.67	0.487	0.912	0.147
Jejunum (g)	20.7	21.8	21.5	21.3	22.0	0.64	0.635	0.892	0.376
Ileum (g)	17.0	18.7	19.0	18.6	19.0	0.89	0.507	0.975	0.392
Ceca (g)	12.9	14.4	14.6	12.9	13.7	0.92	0.561	0.315	0.804
Colon (g)	4.65	5.25	5.85	5.16	4.97	0.34	0.202	0.695	0.277

^a,b,c,d^Means with distinct superscripts within the same row indicate significant differences (*P* < 0.05)

^1^Treatments: CON = standard chicken diet without red grape pomace and no Aloe vera gel in drinking water; GPA1 = standard chicken diet containing 30 g/kg red grape pomace and 1% Aloe vera gel in drinking water; GPA2 = standard chicken diet containing 30 g/kg red grape pomace and 2% Aloe vera gel in drinking water; GPA3 = standard chicken diet containing 30 g/kg red grape pomace and 3% Aloe vera gel in drinking water; GPA4 = standard chicken diet containing 30 g/kg red grape pomace and 4% Aloe vera gel in drinking water

^2^Parameters: FBW = final body weight, HCW = hot carcass weight, CCW = cold carcass weight

^3^SEM: standard error of the mean

### Breast meat quality

Table 6 indicates that there were positive quadratic effects for 24-hour yellowness [*y* = 1.2 (± 1.11) *x* + 12.3 (± 0.16); R^2^ = 0.370, *P* = 0.018], chroma [[*y* = 1.10 (± 1.13) *x* + 12.5 (± 1.17); R^2^ = 0.343, *P* = 0.024], hue angle [*y* = 1.22 (± 1.14) *x* + 12.4 (± 1.15); R^2^ = 0.265; *P* = 0.049], and a negative quadratic effect for 1-hour pH [*y* = 13.5 (± 0.354) – 1.25 (± 0.734); R^2^ = 0.284, *P* = 0.049] in response to AVG doses. There were significant treatment effects on 24-hour L*_24_, b*_24_, and chroma_24_. Birds reared on treatment GPA2 had higher (*P* < 0.05) L*_24_ than those on GPA1 and GPA3 but did not significantly vary with those on CON and GPA4. Breast meat from GPA1 had higher (*P* < 0.05) b*_24_ than those from CON but did not vary from those on GPA3 and GPA4. Breast meat from groups CON and GPA2 showed the least (*P* < 0.05) Chroma_24_ compared to other groups.

**Table 6:** Breast meat quality parameters of densely stocked Ross 308 broiler chickens offered a red grape pomace-containing diet and incremental levels of Aloe vera gel in their drinking water

^2^Parameters	^1^Treatments						Significance		
	CON	GPA1	GPA2	GPA3	GPA4	^3^SEM	*P*-_GLM_	*P*-_Linear_	*P*-_Quadratic_
pH_i_	5.96	5.87	5.85	5.93	5.78	0.07	0.346	0.357	0.049
L*_i_	63.6	62.7	65.8	62.5	65.6	1.18	0.169	0.119	0.528
a*_i_	1.17	1.18	1.21	1.37	0.99	0.19	0.728	0.698	0.256
b*_i_	11.1	13.2	11.6	12.9	14.4	0.88	0.106	0.103	0.141
Chroma_i_	11.1	13.3	11.7	13.0	14.4	0.89	0.111	0.105	0.146
Hue angle_i_	1.46	1.47	1.47	1.46	1.50	0.01	0.274	0.232	0.125
pH_24_	5.94	5.85	5.95	5.91	5.96	0.06	0.653	0.350	0.438
L*_24_	61.6^ab^	57.5^a^	64.2^b^	58.6^a^	61.3^ab^	1.36	0.019	0.487	0.382
a*_24_	2.19	1.60	1.64	2.49	1.50	0.51	0.587	0.767	0.277
b*_24_	11.3^a^	16.3^c^	11.8^ab^	15.6^bc^	16.2^c^	0.98	0.001	0.327	0.018
Chroma_24_	11.5^a^	16.4^c^	12.0^a^	15.9^bc^	16.3^c^	1.02	0.003	0.351	0.024
Hue angle_24_	1.39	1.47	1.43	1.41	1.47	0.03	0.366	0.537	0.049
Cooking loss (%)	16.0	14.1	15.5	12.1	13.8	1.56	0.453	0.849	0.801
Shear force (N)	3.90	5.20	3.70	4.30	4.20	0.47	0.208	0.926	0.324
WHC (%)	80.9	77.1	78.9	78.6	81.6	1.99	0.516	0.109	0.753
Drip loss (%)	2.80	2.90	2.70	3.50	1.50	0.81	0.543	0.392	0.288

^a,b,c^Means with distinct superscripts within the same row indicate significant differences (*P* < 0.05)

^1^Treatments: CON = standard chicken diet without red grape pomace and no Aloe vera gel in drinking water; GPA1 = standard chicken diet containing 30 g/kg red grape pomace and 1% Aloe vera gel in drinking water; GPA2 = standard chicken diet containing 30 g/kg red grape pomace and 2% Aloe vera gel in drinking water; GPA3 = standard chicken diet containing 30 g/kg red grape pomace and 3% Aloe vera gel in drinking water; GPA4 = standard chicken diet containing 30 g/kg red grape pomace and 4% Aloe vera gel in drinking water

^2^Parameters: L* = lightness; a* = redness; b* = yellowness; WHC = water holding capacity

^3^SEM: standard error of the mean

## Discussion

The adoption of nutraceuticals with beneficial antioxidant effects on broiler production, health, and welfare (Egbu et al., 2022; Mnisi et al., 2022) could provide a safe and cost-effective strategy to alleviate SD-induced stress. The cost-effective strategy of these nutraceuticals is premised on the fact that RGP is a waste that is easily accessible and locally available while Aloe vera is highly adaptive and can be planted locally by farmers. This study used a combination of RGP and AVG as potent antioxidant sources to alleviate high SD stress in broiler chickens. There were no notable interaction effects observed between treatment and week (broiler age) on growth performance metrics, suggesting that the efficacy of the birds in utilizing the treatments under high SD was not affected by age. Overall feed intake was not affected by the treatments, which is inconsistent with previous reports, where the use of 25 g/kg grape pomace in broiler diets improved feed intake (Erinle et al., 2022) while the administering of 2.5–5 g/L Aloe vera powder via drinking water reduced feed intake in broilers (Ahmad et al., 2020). However, the simultaneous supplementation with a 30 g RGP /kg diet and 1% AVG in drinking water improved the overall BWG and FCR of the broilers. This indicates that bioactive compounds such as epicatechins and catechins in RGP as well as acemannan, aloesin and emodinin in AVG (Sumi et al., 2019) alleviated high SD stress resulting in improved performance. However, a further increase of AVG in the drinking water between 2 and 4% compromised overall BWG and FCR, indicating that the palatability of the water might have compromised growth metrics. Contrary to our findings, Islam et al. (2017) observed that supplementing broilers with 0.5 and 1% of Aloe vera extract in drinking water had no improvement on growth performance. The differences in these reports indicate that the responses of broilers to the individual or combined use of RGP or AVG depends on the type of production system (including stocking rate), the form of ingredients used (powder, gel, or extract), as well as the dosage levels in diets or drinking water.

Generally, a blood assay is necessary to examine the pathophysiological and nutritional state of birds. In this investigation, the concurrent supplementation with RGP and AVG had a notable impact on erythrocytes. The birds reared on GPA1 exhibited the lowest erythrocyte count, but an observable enhancement in erythrocyte count was noted as the level of AVG increased. The increase in erythrocytes could indicate that the birds synthesized more erythrocytes to cover the shortfalls in nutrient uptake since an increase in AVG (2–4%) did not enhance growth performance. This could explain why the GPA1 group (1% AVG dose) had low erythrocytes because the birds’ growth performance was enhanced. However, it is important to note that the relationship between AVG levels and erythrocyte count followed a negative quadratic trend, with an optimal level of AVG that maximized the erythrocyte count calculated as 2.83%. Quaye et al. (2023) noted higher levels of erythrocyte count in 42-day-old Cobb 500 broilers supplemented with AVG extract at 1% compared to the control groups. The authors attributed the increase to the erythropoietin effects of AVG on hemopoietic cells in the bone marrow, which can improve erythrocyte production (Iji et al., 2010). Contrary, Tariq et al. (2014) observed no change in erythrocyte levels in 35-day-old Japanese quail, whose diets were supplemented with Aloe vera and clove (*Syzigium aromaticum*). Jonathan et al. (2021) also observed linear increases in erythrocytes, MCH, and eosinophils when 45 g/kg of RGP was supplemented in diets of Hy-line silver-brown cockerels, further indicating that higher AVG doses in this study compromised erythrocyte counts. Positive quadratic effects were noted for MCH as the level of AVG increased, which is a measure of the average amount of hemoglobin present in the red blood cells (Schaer et al., 2013). Although it was not influenced by the treatments, hemoglobin is the protein responsible for carrying oxygen to various tissues and organs in the body and is essential for normal physiological functions (Schaer et al., 2013). When the level of AVG was increased, the MCH levels initially showed an increase before declining. This observation corroborates the negative quadratic response for erythrocytes. Basophils are a type of white blood cell involved in the immune response and inflammatory processes (Voehringer, 2017). The positive quadratic effect on basophils suggests that initially, as the level of AVG increased, there was an increase in basophil count. However, doses of AVG beyond 2.92% caused a decline in basophil count, which could be due to the biological effects of AVG on the immune system. Indeed, AVG contains polysaccharides, flavonoids, and anthraquinones that possess immunomodulatory properties (Ahmad et al., 2020). Thus, administering 1% AVG via drinking water might have stimulated the activation of immune cells and the release of chemical mediators involved in immune responses, leading to an increase in basophil count. However, AVG levels beyond 2% in the drinking water could have suppressed immune responses resulting in a decrease in basophil count.

The simultaneous treatment of the densely stocked birds with RGP and AVG influenced SDMA, phosphorus, and amylase. Amylase is an enzyme that aids in the digestion of carbohydrates while SDMA is a biomarker used to assess kidney function in animals (Sargent et al., 2021). Phosphorus plays a major role in various physiological processes, including bone health, energy metabolism, and DNA synthesis. Thus, the increase observed in SDMA, phosphorus, and amylase levels with AVG doses indicates decreased kidney function, disrupted mineral metabolism, and pancreatic dysfunction, respectively. This result cannot be attributed to the bioactivities of acemannan, aloesin, emodin and aloin in AVG (Sumi et al., 2019) as well as procyanidins, epicatechin-3-O-gallate, epicatechins, and catechins in RGP (Rockenbach et al., 2011) because the RGP and AVG groups did not show any variation when compared to the control group. Contrary, Singh et al. (2013) and Sharma et al. (2018) found that the supplementation of broiler chickens with dietary Aloe vera did not influence SDMA, phosphorus, and amylase. The inconsistent results could be because the Aloe vera was combined with RGP in the current study. Higher doses of AVG could have interfered with the taste and palatability of the water resulting in poor utilization of AVG bioactive compounds to positively stimulate physiological changes.

Establishing a balanced relationship between SD and welfare is important to boost societal acceptance of poultry products. Leg weakness and dermatitis represent two major welfare concerns for broiler chickens, which can compromise welfare (Bradshaw et al., 2002). The concurrent treatment of the broilers with RGP and AVG influenced gait scores where the GPA2 group had the lowest mean score (18.70), and the CON group showed the highest mean score (31.20). This result implies that the simultaneous treatment of densely stocked chickens with a 30 g RPG /kg diet and incremental levels (1–4%) of AVG had a positive synergistic impact on the gait score. Birds reared under high stocking densities usually experience suboptimal growth, lesions, and walking difficulties, especially at the finisher phase (BenSassi et al., 2019). This is the reason gait score and LTL assessments were determined at the end of the finisher phase, considering that the birds may be more susceptible to locomotive disorders due to heavier body weights (Swiatkiewicz et al., 2017). The GPA4 group had the longest LTL (529.87 s) while the CON group had the shortest (159.03 s) times, further suggesting that the concurrent treatment of densely stocked birds with RGP and AVG reduced their susceptibility to locomotive disorders. This finding can be ascribed to the polyphenols present in these nutraceuticals, which modulate inflammation, oxidative stress, and cellular damage, thus improving the overall leg health and mobility of the chickens. Good feather coverage is said to reflect optimal feed utilization efficiency (Leeson and Walsh, 2004). Thus, the lack of treatment effects on feather score shows that the supplementation with RGP and AVG did not provide additional benefits on feather coverage.

Carcass yield is used to assess the performance and economic profitability of broiler production. The present results showed that the simultaneous treatment of densely stocked birds with a 30 g RPG /kg diet and 1% AVG had a synergistic positive impact on finisher weight than the CON group. This finding was attributed to the growth-stimulating properties of RGP and AVG bioactive compounds. This finding contradicts those of Mehala and Moorthy (2008) and Tariq et al. (2014), who found that carcass yield and breast weight were influenced by the supplementation with Aloe vera and *Curcuma longa* in broilers as well as Aloe vera and clove (*Syzigium aromaticum*) powder in Japanese quail, respectively. The contradicting results might be due to the species differences, the type of feed additives that were combined with the Aloe vera, and the route of administration. Nonetheless, the impacts of RGP and AVG were not observed on the internal organs. This implies that the concurrent treatment of the birds with RGP and AVG did not compromise gastrointestinal tract and internal organ development. This could explain the observed similarities among the treatments for serum ALT and ALKT, which are liver enzymes known to increase in response to liver damage (Jonathan et al., 2021).

The general acceptance of meat and meat products by consumers depends on appearance, juiciness, and tenderness. A negative quadratic effect was observed on initial breast meat pH as AVG levels increased, which could be attributed to the antimicrobial properties of acemannan, aloesin, aloin, and emodin in AVG (Sumi et al., 2019). At slaughter, glycogen is converted into lactic acid, and ultimately, the accumulation of lactate leads to a drop in meat pH (Alnahhas et al., 2014). Therefore, the antimicrobial activities in AVG might have destroyed other microbes and enhanced the proliferation of lactate bacteria (Khan et al., 2022). However, the rate of pH decline and the ultimate pH can vary based on species, pre-slaughter handling, stress levels, temperature, and muscle conditions. Thus, proper handling and chilling of the carcass after slaughter are important to ensure the meat reaches the desired pH levels for quality and food safety considerations.

A positive quadratic effect was recorded for meat yellowness, chroma, and hue angle measured 24 h post-mortem. The AVG is derived from the internal part of the Aloe vera leaves and is typically colorless and does not contain any natural pigments that would significantly alter the color of meat (Jeyaram, 2022). Thus, the observed color changes could have been a result of handling or storage and some pigments (anthocyanins) in RGP (Sharma et al., 2021). This claim could be supported by the higher breast meat yellowness and chroma measured 24 h post-mortem in the GPA1, GPA3, and GPA4 groups than the CON group. The water loss or retention in meat is primarily influenced by the tissue’s pH level with lower pH leading to increased water loss (Alnahhas et al., 2014). The absence of any treatment effects on ultimate meat pH could explain the lack of changes in WHC, drip loss, cooking loss, and shear force. This shows that simultaneous treatment of densely stocked birds with RGP and AVG did not negatively impact breast meat quality.

## Conclusion

The concurrent treatment of densely stocked broilers with a 30 g red grape pomace /kg diet and 1% Aloe vera gel in drinking water improved growth metrics and finisher body weight. The treatments also altered some health indicators, gait scores, and meat quality attributes. Higher doses of Aloe vera gel beyond 1% did not enhance performance and physiological traits of the broilers indicating that higher levels do not ameliorate high stocking density stress. Future studies could assess the effectiveness of red grape pomace and Aloe vera nutraceuticals using different modes of application to this study. However, it is worth noting that the nutraceutical composition of these plants varies with growth environment, processing, and type of variety.

## Data Availability

The data for this study is available on request from the corresponding author.
